# Early mortality risk stratification in childhood bacterial meningitis using cerebrospinal fluid glucose and protein levels and their combinations: A multicontinental cohort study

**DOI:** 10.1371/journal.pone.0351306

**Published:** 2026-06-09

**Authors:** Markku Kallio, Tuula Pelkonen, Irmeli Roine, Heikki Peltola

**Affiliations:** 1 New Children’s Hospital, University of Helsinki and Helsinki University Hospital, Finland; 2 Hospital Pediátrico David Bernardino, Luanda, Angola; 3 New Children’s Hospital, Pediatric Research Center, Helsinki, Finland; 4 Faculty of Medicine, University Diego Portales, Santiago, Chile; University of Sri Jayewardenepura, SRI LANKA

## Abstract

**Background:**

Bacterial meningitis remains a major cause of death and neurological disability in children worldwide, particularly in low-resource settings where access to intensive care is limited. Cerebrospinal fluid (CSF) glucose, protein, and leukocyte concentrations are routinely measured at hospital presentation, yet their potential for early risk stratification – individually or in combination – has not been clearly defined. Because mortality risk is often difficult to assess at admission, identifying simple CSF-based thresholds that help clinicians recognize high-risk patients could improve triage and management globally.

**Methods and findings:**

We evaluated CSF glucose, protein and leukocyte levels in 1598 children with bacteriologically confirmed bacterial meningitis across six countries in Latin America, Angola, and Finland to determine their individual and combined associations with mortality and neurological outcome. CSF glucose, protein, and leukocyte counts were measured at admission and examined in relation to in-hospital mortality and neurological outcome at discharge, as assessed by the Glasgow Outcome Scale. CSF glucose and protein concentrations were categorized into five clinically interpretable groups. Mortality increased progressively with decreasing CSF glucose (from 8% to 29%) and with increasing protein concentrations (from 5% to 23%). Children with both low CSF glucose (<10 mg/dL) and high CSF protein (≥200 mg/dL) had a mortality rate of 27% compared with 1% among those with normal CSF glucose and protein. A CSF leukocyte count <500/µL was also associated with increased mortality. Lower CSF glucose (ρ = 0.24; P < .001) and higher protein (ρ = 0.15; P < .001) were both associated with poorer neurological outcomes.

**Conclusions:**

Routine CSF markers available at the time of hospital admission provide prognostic information that supports risk stratification in pediatric bacterial meningitis. Simple CSF-based thresholds—particularly when CSF glucose and protein are interpreted in combination—may help identify high-risk children early and guide clinical prioritization in settings with limited critical care capacity.

## Introduction

Bacterial meningitis remains a severe and life-threatening disease in children, with substantial mortality and neurological sequelae despite global vaccination programs and advances in antimicrobial therapy [1,2]. The burden is greatest in low- and middle-income countries, where delays in diagnosis and limited access to intensive care contribute to high case fatality rates. Early risk assessment is crucial for guiding management and resource allocation, yet the prognostic information embedded in routine cerebrospinal fluid (CSF) measurements – particularly glucose and protein – has not been fully utilized in clinical practice. Clinical presentation of bacterial meningitis is variable: although the classical triad of fever, headache, and vomiting is well recognized, many children present with nonspecific findings, making early diagnosis challenging [3–6]. Consequently, lumbar puncture remains essential for early evaluation in all suspected cases.

Because the causative pathogen is seldom identified at admission except by Gram stain, clinicians rely on routine CSF analyses to guide diagnosis and early treatment decisions. In bacterial meningitis, CSF glucose is typically reduced, protein is elevated, and leukocyte counts are increased, reflecting inflammatory processes within the central nervous system [7,8]. However, laboratory findings vary widely between patients. Approximately 6% of children with culture-confirmed bacterial meningitis have normal CSF leukocyte counts, and lymphocyte predominance is observed in up to 10% of cases [9]. Such variability limits the diagnostic and prognostic value of CSF leukocyte counts alone.

CSF glucose decreases due to blood-brain barrier disruption, increased glycolytic activity of inflammatory cells, and bacterial consumption of glucose [6,8]. In contrast, elevated CSF protein reflects increased blood-brain barrier permeability in severe inflammation and has been associated with poor outcomes [10–12]. Although CSF glucose and protein are not direct inflammatory biomarkers, they are universally available, low-cost indicators of central nervous system involvement – even in settings lacking advanced laboratory capacity. Several prognostic models have been developed to improve risk stratification in meningitis [13–17], including the Bacterial Meningitis Score [18] and the Meningitis Score for Emergencies [19]. However, these tools are designed primarily to distinguish bacterial from viral meningitis rather than to predict mortality, and their applicability in resource-limited settings is restricted by dependence on laboratory parameters that are not routinely available. Importantly, CSF glucose – long recognized as a marker of poor prognosis – is not explicitly incorporated into commonly used prognostic tools [5].

Over the years, our group has conducted five large prospective studies of childhood bacterial meningitis across three continents – six countries in Latin America, Angola, and Finland – yielding a clinically diverse dataset that reflects real-world conditions in both low- and high-resource settings. This dataset enables evaluation of two key clinical questions with direct global relevance: [1] Can mortality risk in childhood bacterial meningitis be predicted using routine CSF measurements at admission to hospital, based on clinically interpretable thresholds for glucose, protein, and leukocytes? and [2] Do combinations of these CSF markers identify children at highest risk of death?

## Methods

### Patients and data collection

We conducted five prospective bacterial meningitis trials in between 1984 and 2017 in Finland [20], 6 countries in Latin America [21], and Angola [22], with detailed descriptions published elsewhere [23]. Number of patients in Finland (n = 334), in Angola (n = 721), and in Latin American countries (n = 543): Argentina (n = 131), Brazil (n = 14), Dominican Republic (n = 106), Ecuador (n = 112), Venezuela (n = 108), Paraguay (n = 72). The data used in this study were combined into a single dataset on 15/9/2020. Bacterial meningitis was confirmed if any of the following criteria were met: positive CSF culture; compatible clinical features with a positive blood culture; positive CSF latex agglutination test; positive CSF Gram stain; or positive CSF polymerase chain reaction [20–23]. Exclusion criteria included age younger than 2 months, trauma, intracranial shunt, preexisting hearing impairment or neurologic disease, and use of immunosuppressive medication. Patients diagnosed with encephalitis or tuberculous meningitis were excluded from the study. On admission, the attending pediatrician performed a physical examination and lumbar puncture, ordered relevant tests, and completed a standardized questionnaire used across all trials. The child’s presenting condition was graded with the age-adjusted Glasgow Coma Scale (range 3–15) [24].

At hospital discharge, a clinical evaluation was performed with special attention to neurologic and audiologic outcomes. Outcomes were assessed using the modified Glasgow Outcome Score (range, 1–5), with 1 indicating uneventful recovery and 5 indicating death. Intermediate categories included survival with mild hearing deficit (better-ear threshold, 41–79 dB), deafness (≥80 dB), and mild or severe neurologic sequelae. Mild sequelae included hemiparesis, monoparesis, psychomotor retardation, or ataxia, whereas severe sequelae included blindness, quadriplegia/paresis, hydrocephalus requiring a shunt, or severe psychomotor retardation.

CSF and blood glucose concentrations were measured, CSF protein levels assessed, CSF leukocytes counted, and bacteria identified at each institution using standard methods. Normal reference values were defined as CSF glucose concentration ≥40 mg/dL, CSF protein concentration <60 mg/dL, CSF leukocytes <5/mm³, and a CSF-to-blood glucose ratio of 0.4. The large number of bacteriologically confirmed meningitis cases (n = 1598) allowed analysis of CSF variables by dividing concentrations into 5 clinically practical and easily interpretable groups, ensuring sufficient numbers in each category for trend analyses. The objective was to determine whether simple, clinically interpretable thresholds could be identified to help clinicians assess mortality risk. CSF glucose was categorized as normal (≥40 mg/dL), 30 to <40 mg/dL, 20 to <30 mg/dL, 10 to <20 mg/dL, and <10 mg/dL. CSF protein was categorized as <60 mg/dL, 60–99 mg/dL, 100–199 mg/dL, 200 to <299 mg/dL, and ≥300 mg/dL. CSF leukocytes were categorized as <100/mm³, 100–499/mm³, 500–999/mm³, 1000–1999/mm³, and ≥2000/mm³. Data from daily completed questionnaires were collected and analyzed in the original studies, and all data were subsequently combined and analyzed in Finland [20–23].

### Ethics approval and consent to participate

The Ethics Committee of Luanda Children’s Hospital approved the studies conducted in Angola, and the hospital boards approved the studies in Latin America and Finland (1984–2017). Following the initiation of international clinical trial registration, the 2 Angolan studies were registered (ISRCTN62824827; April 10, 2005, and NCT01540838; February 29, 2012). Patients were enrolled only after written or oral informed consent was obtained from their legal guardian; for illiterate guardians, a fingerprint served as consent. All methods were conducted in accordance with the Declaration of Helsinki.

### Statistical analysis

Data analysis was performed using JMP Pro 15.0 (SAS Institute Inc) for MacOS. Descriptive data are presented as counts with percentages or medians with interquartile ranges (IQRs), as appropriate. Differences in baseline characteristics between groups were assessed using the Wilcoxon or Kruskal-Wallis tests, χ² tests, or the Mann-Whitney test, depending on data type. Associations between ordinal categorical exposures and binary outcomes were evaluated with the Cochran-Armitage trend test, which detects trends across ordered groups in a binary response variable. Glasgow Outcome Score results were compared with CSF glucose, protein, and leukocyte counts (continuous data) using Spearman correlation. Nominal logistic regression analysis was used to evaluate survival outcomes, with odds ratios and 95% CIs calculated for predictor variables. The Effect Likelihood Ratio test was applied to the final model, demonstrating significant contributions from CSF glucose, CSF protein and altered consciousness. Model performance was evaluated using a Receiver Operating Characteristic (ROC) curve, with the Area Under the Curve (AUC) indicating a moderate ability to differentiate between survival and mortality.

## Results

A total of 721 children from Angola, 543 from Latin America, and 334 from Finland comprised the series of 1598 cases of bacterial meningitis caused by Haemophilus influenzae (n = 668), Streptococcus pneumoniae (n = 506), Neisseria meningitidis (n = 285), or other bacteria (n = 139). The etiology was determined by CSF culture in 1225 cases (77%), CSF latex agglutination test in 97 (6%), CSF Gram stain in 124 (8%), CSF polymerase chain reaction in 98 (6%), and blood culture in 54 (3%). Males accounted for 862 cases (55%) and females for 696 cases (45%). The median age was 1.2 years (IQR, 0.6–3.2) for boys and 1.0 years (IQR, 0.5–2.8) for girls. Country-specific laboratory and clinical variables are presented in Table 1.

**Table 1:** Patient characteristics and cerebrospinal fluid (CSF) values at admission and during illness. Data are presented as median values; percentages or interquartile ranges (25th - 75th percentile) are shown in parentheses, as appropriate.

	Whole series	Finland	Latin America	Angola
**At arrival**				
Age – years	1.1 (0.5-3.1)	1.8 (0.8-3.4)	0.8 (0.5-2.6)	1.0 (0.5-2.9)
Ill before arrival – days	3 (2 –5)	1 (1 –3)	2 (2–3)	4 (3 –7)
Pretreatment antibiotics (%)	502/1454 (35%)	58/323 (18%)	169/494 (34%)	275/637 (43%)
Decreased level of consciousness (Glasgow coma score <15) (%)	1112/1473 (75%)	263/325 (79%)	401/512 (78%)	448/636 (72%)
Seizures before / at arrival (%)	585/1515 (39%)	60/328 (18%)	182/513 (35%)	343/674 (51%)
Axillary temperature °C	38.0 (37.0-39.0)	39.0 (38.3-39.7)	38.0 (37.0-38.6)	37.7 (37.0-38.5)
CRP (mg/L)	158 (91-161)	130 (78-195)	152 (73-169)	161 (110-161)
Blood leucocytes (10^3^/µL)	14.8 (9.1-21.0)	13.6 (8.5-20.0)	14.9 (9.0-21.1)	15.3 (9.8-22.1)
CSF Glucose (mg/dL)				
Survived	16 (7–37)^a^	32 (13-60)	14 (5–33)^a^	13 (6–24)^b^
Died	10 (5 –17)	7 (2-69)	6 (3 –15)	10 (6 –17)
CSF Protein (mg/dL)				
Survived	160 (90–250)^a^	160 (90–240)^b^	150 (90–250)^b^	190 (120–260)^c^
Died	230 (170-370)	390 (190-480)	250 (150-470)	210 (180-280)
CSF Leucocytes (/mm^3^)				
Survived	2010 (700–5700)^c^	3030 (1270–6900)^c^	1960 (680–6200)^c^	1500 (430–3500)^c^
Died	1330 (320-4470)	2400 (480-8300)	1560 (400-6830)	1250 (290-3790)
**During illness course**				
Days in hospital	10 (8 –12)	11 (9 –11)	8 (7 –11)	11 (9 –17)
Deafness ^d^ (%)	110/1034 (11%)	22/271 (8%)	45/375 (12%)	43/388 (11%)
Neurological sequelae (%)	380/1194 (32%)	56/322 (17%)	133/450 (29%)	191/422 (45%)

a = p < 0.001 b = p < 0.01 c = p < 0.05 These differences are between survived and died patients

d = better ear's hearing threshold ≥80 dB

### CSF glucose and survival

**Fig 1:**
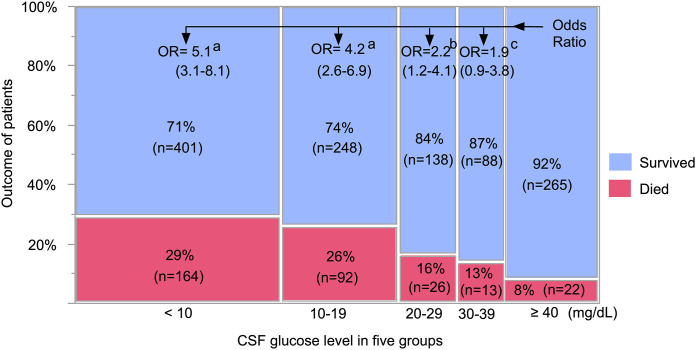
Survival vs death in children with bacterial meningitis by cerebrospinal fluid (CSF) glucose concentration at hospital admission. Odds ratios compare normal glucose values (≥40 mg/dL) with lower concentrations categorized into 4 groups. a = p < 0.001 b = p < 0.01 c = p < 0.05.

Lower CSF glucose concentrations were associated with higher mortality (Fig 1). A significant linear trend was observed between decreasing CSF glucose levels and increased mortality (Cochran-Armitage trend test, P < .001; χ² test, P < .001). For overall outcomes at discharge, measured by the Glasgow Outcome Score, CSF glucose as a continuous variable correlated with outcome (Spearman ρ = 0.24; P < .001).

Because the study period was long, during which vaccines were introduced and treatment practices changed, we compared outcomes across each decade. The prognostic value of low CSF glucose and high CSF protein for mortality remained consistent across all time periods. Similarly, the findings were unchanged when analyses were stratified by the major bacterial pathogens or patients with and without antibiotic pretreatment.

### CSF protein and survival

**Fig 2:**
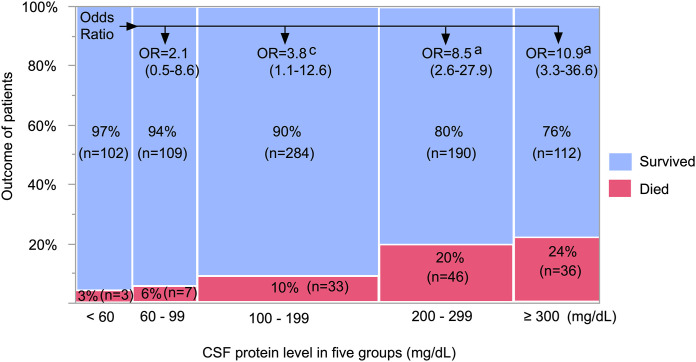
Survival vs death in children with bacterial meningitis by cerebrospinal fluid (CSF) protein concentration at hospital admission. Odds ratios compare normal protein values (<60 mg/dL) with higher concentrations, categorized into 4 groups. a = p<0.001 c = p<0.05.

Higher CSF protein concentrations were significantly associated with increased mortality (Fig 2). A significant linear trend was observed across protein categories (Cochran-Armitage trend test, P < .001; χ² test, P < .001). For overall outcomes at discharge, measured by the Glasgow Outcome Score, CSF protein as a continuous variable correlated with outcome (Spearman ρ = 0.16; P < .001).

### CSF leucocyte and survival

CSF leukocyte count, one of the most commonly used diagnostic indices in bacterial meningitis, was categorized into five groups. A low CSF leukocyte count was associated with higher mortality. Children admitted with counts <500/mm³ had approximately a 50% greater risk of death compared with those with higher counts. However, once the leukocyte count exceeded 500/mm³, no additional prognostic value was observed, whether the count was 1000/mm³, 2000/mm³, or higher (Cochran-Armitage trend test, P = non-significant). For overall outcomes at discharge, measured by the Glasgow Outcome Score, CSF leukocyte count as a continuous variable correlated with outcome (Spearman ρ = 0.13; P < .01).

### The CSF-to-blood glucose ratio and survival

The CSF-to-blood glucose ratio was less strongly associated with mortality than CSF glucose concentration alone, although the trend remained significant (Cochran-Armitage trend test, P < .01; χ² test, P < .05). When the ratio was only moderately decreased (≥0.20), the trend was less evident.

### CSF glucose and survival in *Streptococcus pneumoniae* meningitis

**Fig 3:**
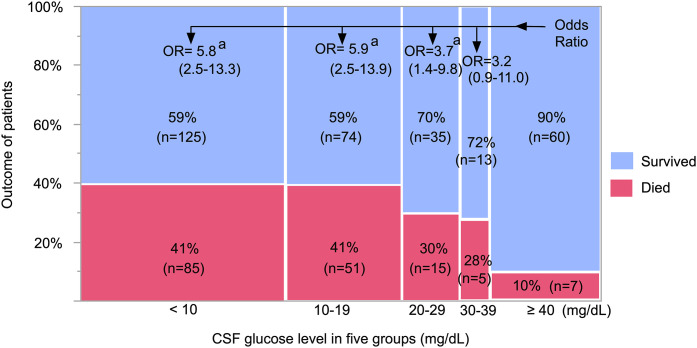
Survival vs death in children with *Streptococcus pneumoniae* meningitis by cerebrospinal fluid (CSF) glucose concentration at hospital admission. Odds ratios compare normal CSF glucose values (≥40 mg/dL) with lower concentrations, categorized into 4 groups. a = p < 0.001.

Because the incidence of Haemophilus influenzae and Neisseria meningitidis meningitis has markedly decreased with vaccination, we analyzed the association between CSF glucose levels and mortality in Streptococcus pneumoniae meningitis. Mortality was significantly higher at lower CSF glucose levels compared with higher levels (Fig 3). A significant linear trend was observed between decreasing CSF glucose concentrations and increased mortality (Cochran-Armitage trend test, P < .001; χ² test, P < .001).

### Survival in patients with Markedly Low CSF Glucose and High CSF Protein

**Fig 4:**
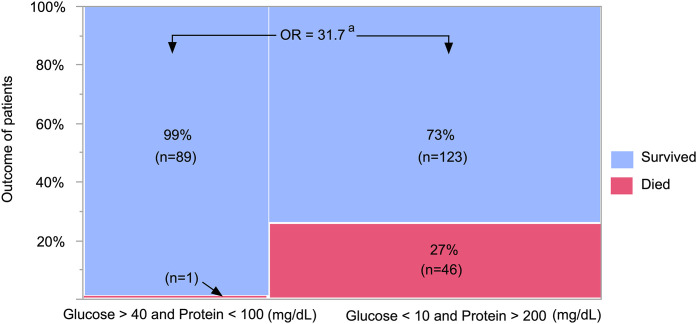
Survival vs death in children with bacterial meningitis comparing those with normal cerebrospinal fluid (CSF) glucose (>40 mg/dL) and CSF protein (<60 mg/dL) values with those with very low CSF glucose (<10 mg/dL) and very high CSF protein (>200 mg/dL) values. Odds ratio compares these 2 groups. a = p < 0.001.

Because both reduced CSF glucose and elevated CSF protein were strongly associated with mortality, we evaluated their combined prognostic effect by comparing children with markedly abnormal values to those with both parameters within the normal range (Fig 4). Mortality was 27% (46 of 169) among patients with concomitantly low CSF glucose <10 mg/dL and high CSF protein >200 mg/dL values, compared with 1% (1 of 90) among patients with normal CSF glucose and CSF protein values (χ² = 35.1; P < .001; Odds ratio, 31.7; 95% CI, 4.4–237.0).

### The survival of patients with decreased level of consciousness (Glasgow coma scale under 15) and its association with varying CSF glucose or CSF protein levels

A Glasgow coma scale under 15 upon hospital admission correlates with a poorer prognosis in bacterial meningitis. In our dataset, approximately 75% of patients presented to the emergency department with a GCS < 15, enabling us to study how CSF glucose and protein levels behaved in these patients in relation to survival. Patients with a GCS < 15, but normal or near to normal CSF glucose (>30 mg/dL) and protein levels (<100 mg/dL) had a better prognosis compared to the other groups. Conversely, if patients with a GCS < 15, had low CSF glucose (<10 mg/dL) or high CSF protein (>200 mg/dL), survival was significantly poorer.

### Predictors of survival in bacterial meningitis

To investigate predictors of survival in bacterial meningitis, a nominal logistic regression analysis was conducted using CSF glucose, CSF protein, altered consciousness, age, sex, pulse, pretreatment antibiotics, study region and duration of illness at hospital admission as explanatory variables. Age, sex, pulse, pretreatment antibiotics, study region and duration of illness were initially included in the model but were found to be non-significant (p > 0.05) and were subsequently excluded from the final analysis. In the final model, the Effect Likelihood Ratio -test indicated significant contributions from CSF glucose (Chi Square = 23.7, p < 0.001), CSF protein (Chi Square = 22.1, p < 0.001) and altered consciousness (GCS < 15) (Chi Square = 14.2, p < 0.001). The model’s discriminatory ability was assessed using the Receiver Operating Characteristic (ROC) curve, which yielded an Area Under the Curve (AUC) of 0.74, indicating a moderate ability to distinguish between survival and mortality outcomes. The ROC analysis was based on the final logistic regression model, which included the significant predictors identified in the Effect Likelihood Ratio test: CSF glucose, CSF protein, altered consciousness.

## Discussion

CSF analysis is central to the diagnosis and management of bacterial meningitis, yet its potential contribution to early prognostic assessment remains underexplored. In this multicontinental cohort of 1598 children with bacteriologically confirmed meningitis, we found a clear linear association between decreasing CSF glucose, increasing CSF protein, and mortality. These findings are consistent with earlier observations linking low CSF glucose to adverse outcomes [6,10,25,26], but our study extends previous work by demonstrating that mortality risk increases progressively across glucose and protein strata rather than at a single critical threshold. Importantly, children with combined low CSF glucose and high CSF protein were at markedly increased risk of death, suggesting that simultaneous impairment of glucose transport and blood-brain barrier integrity reflects severe meningeal inflammation and metabolic distress. Because CSF glucose and protein are among the first laboratory results available at admission – even in resource-limited settings – these findings have direct clinical relevance, providing simple parameters that may support early risk stratification and guide triage decisions before etiological confirmation. The associations between these CSF abnormalities and poorer neurological outcomes at discharge further highlight their value as early indicators of disease severity.

Glucose is the primary metabolic substrate for the brain and is transported into the cerebrospinal fluid through facilitated diffusion across the blood-brain barrier. In bacterial meningitis, the inflammatory response disrupts blood-brain barrier function and impairs glucose transport, while activated leukocytes and bacteria increase glycolytic consumption of glucose within the subarachnoid space [27]. These mechanisms explain the progressive decline in CSF glucose observed in our study and its strong association with mortality. We found that severely low CSF glucose levels, particularly below 10 mg/dL, were associated with markedly increased risk of death. The flattening of the risk gradient beyond these thresholds (<10 mg/dL and <20 mg/dL) suggests a point of metabolic failure, at which compensatory mechanisms are insufficient to maintain cerebral energy supply. Low CSF glucose has previously been associated not only with mortality but also with neurological complications such as sensorineural hearing loss [28], further supporting its role as a marker of severe disease.

Although measurement of the cerebrospinal fluid (CSF)-to-blood glucose ratio is traditionally recommended to aid in the diagnosis of bacterial meningitis [8,29,30]. In our cohort, the ratio was a less sensitive predictor of survival than CSF glucose or protein concentrations alone. In routine practice, its use is constrained by practical limitations: blood and CSF samples must be obtained simultaneously, which is often not feasible in emergency settings, particularly after the initiation of intravenous fluids. In many of our patients, blood glucose values were influenced by glucose-containing maintenance fluids that were initiated when the child presented to the emergency department, which distorted the (CSF)-to-blood glucose ratio and reduced its discriminative value. Furthermore, when CSF glucose levels are severely reduced, the ratio remains low regardless of blood glucose concentration, reducing the clinical relevance of the denominator.

Under physiological conditions, the CSF contains only small amounts of plasma-derived proteins that cross the blood-brain barrier through selective filtration. In bacterial meningitis, inflammatory injury disrupts endothelial junctions and increases barrier permeability, allowing excess serum proteins to enter the subarachnoid space. Consistent with previous reports linking elevated CSF protein to adverse outcomes [10,17,31,32], we found that the risk of death increased progressively with rising protein concentrations. Markedly elevated CSF protein levels (>200 mg/dL), particularly those exceeding 300 mg/dL, were associated with substantially worse prognosis compared with only mildly elevated values. This pattern likely reflects the extent of blood-brain barrier disruption and the severity of the inflammatory response within the central nervous system.

The combination of CSF glucose and protein provided substantially greater prognostic discrimination than either marker alone. Mortality in bacterial meningitis was low among children with both markers within the normal range, whereas approximately one-third of those with severely low CSF glucose (<10 mg/dL) and markedly elevated protein (>300 mg/dL) died. These findings suggest that concurrent failure of glucose transport and significant blood-brain barrier disruption reflect severe inflammatory pathology and high early mortality risk. In contrast, although CSF leukocyte count remains essential for diagnosis, its prognostic value was limited. Leukocyte counts below 500/mm³ were associated with increased mortality, consistent with impaired host immune response, but values above this threshold did not differentiate survivors from non-survivors, indicating a ceiling effect. In severe infections such as meningitis with septic shock, CSF leukocyte counts may be unexpectedly low, reflecting overwhelming infection and impaired host response. Low CSF leukocyte counts have been shown to be independently associated with adverse outcomes in bacterial meningitis [6,33]

In clinical practice, mortality risk in childhood bacterial meningitis is often difficult to assess at the time of admission, particularly in settings where clinical severity scores with advanced laboratory testing are unavailable. Our findings show that simple combinations of routine CSF measurements – glucose and protein – can identify children at high risk of death using thresholds that are immediately interpretable by clinicians. These results support incorporation of CSF glucose and protein combinations into early risk assessment and triage protocols and may provide a foundation for pragmatic severity classification in future clinical trials. Because these measurements are already part of standard diagnostic evaluation worldwide, they could be implemented rapidly without additional cost, training, or equipment. Further studies are needed to evaluate whether CSF-based thresholds can guide intensified monitoring or adjunctive therapy and to determine their prognostic value in culture-negative disease. Tools that leverage existing diagnostics rather than requiring new technologies have potential for global impact, particularly in low-resource settings where most deaths from bacterial meningitis occur and timely triage remains a challenge.

This study has several strengths. It includes one of the largest prospectively collected cohorts of childhood bacterial meningitis with bacteriological confirmation and standardized data collection across three continents. The multinational design captures a broad clinical spectrum from both high-resource and low-resource settings, increasing the external validity of the findings. Importantly, the prognostic assessment was based on routine CSF parameters available at admission, enhancing clinical applicability even in hospitals with limited diagnostic resources.

This study also has limitations. Differences in socioeconomic and clinical conditions across the 3 continents may have influenced outcomes. We attempted to mitigate the long study duration by focusing on admission CSF findings, which were independent of treatment protocols. Patient care in all hospitals included supportive measures such as oxygen and fluid therapy, and laboratory practices for CSF analysis were consistent across sites and over time. Focusing on bacteriologically confirmed cases enhances reliability but may constrain generalizability to children with suspected meningitis and abnormal CSF findings without confirmed bacterial etiology. The prognostic value of CSF glucose and protein in culture-negative cases remains uncertain, highlighting the need for further research in this population, particularly in resource-limited settings where culture results may be unavailable or delayed.

## Conclusion

This multicontinental analysis shows that simple cerebrospinal fluid measurements obtained at admission can stratify mortality risk in childhood bacterial meningitis without reliance on advanced diagnostics. These findings support the development of pragmatic, severity-based triage strategies using existing laboratory resources. Validation of these thresholds in prospective studies, including children with culture-negative meningitis, will be essential to guide future clinical application and to reduce preventable deaths in high-burden regions.

We confirm that:

• All authors have made substantial contributions to the work,

• All authors have approved the final manuscript,

• The manuscript has not been published previously and is not under consideration by any other journal,

• There are no conflicts of interest to declare
