# Supplementary material for: Early mortality risk stratification in childhood bacterial meningitis using cerebrospinal fluid glucose and protein levels and their combinations: A multicontinental cohort study
Source: PLoS One. 2026 Jun 9;21(6):e0351306. doi: 10.1371/journal.pone.0351306 (PMC13249170; doi:10.1371/journal.pone.0351306)
Supplement: Attachment — Submitted filename: PLOS ONE- review.docx [file pone.0351306.s001.docx]

PLOS ONE

Early mortality risk stratification in childhood bacterial meningitis using cerebrospinal fluid glucose and protein levels and their combinations: a multicontinental cohort study

| Manuscript Number: | PONE-D-25-60645 |
| --- | --- |

1. Recommendation (Answer options: Accept, Minor Revision, Major Revision, Reject)
   a. Minor revisions
2. Is the manuscript technically sound, and do the data support the conclusions? (Answer options: Yes, No, Partly)
   a. Partly
3. Has the statistical analysis been performed appropriately and rigorously? (Answer options: Yes, No, I don’t know, N/A)
   a. Yes
4. Does the manuscript adhere to the PLOS Data Policy? Additional details can be found at <http://journals.plos.org/plosone/s/materials-and-software-sharing>. (Answer options: Yes, No)
   a. No
5. Is the manuscript presented in an intelligible fashion and written in standard English? (Answer options: Yes, No)
   a. Yes
6. Review Comments to the Author (minimum 200 characters)

I congratulate the authours for the excellent study which is clinically oriented and very useful for day to day practise. It is a large study with more than 1500 patients and is multi-centre. Here are some minor concerns which I would like to raise.

1. Description of Study Population (Latin America)

It would improve clarity to explicitly list the individual Latin American countries included in the study and the number of participants recruited from each country. This will help readers better understand the geographic distribution and heterogeneity of the cohort.

2. Very Long Study Period (1984–2017)

The study spans 33 years, during which substantial changes have occurred in:

- Epidemiology of pathogens due to vaccination (Hib, pneumococcal vaccines)
- Antibiotic regimens and resistance patterns
- Supportive and intensive care management
- Diagnostic modalities (PCR availability, improved microbiology techniques)

Pooling all data across this period may introduce temporal bias unless the authors demonstrate that pathogen distribution, management strategies, and outcomes were comparable across eras. A subgroup analysis by time period (e.g., pre- and post-vaccine eras) is strongly recommended.

3. Inclusion of Encephalitis Cases

Were patients with encephalitis included in this cohort? If so, please clarify the diagnostic criteria and describe their number and characteristics. If not, this should be clearly stated.

4. Inclusion of Tuberculous Meningitis

Were patients with tuberculous meningitis included? If yes, please provide details. If excluded, this should be explicitly mentioned in the methods.

5. Breakdown of the “Other Bacteria” Category

The “other” pathogen group is heterogeneous. Certain organisms (e.g., *Listeria monocytogenes*, *Mycoplasma pneumoniae*) may cause lymphocytic predominance with less pronounced CSF glucose reduction or protein elevation but can still result in severe outcomes.

A detailed breakdown of pathogens within the “other” category is essential. If numbers are small and heterogeneity substantial, consider:

- A pathogen-specific sub-analysis
- Or restricting conclusions to the major pathogens (*H. influenzae*, *S. pneumoniae*, *N. meningitidis*)

6. Description of Treatment Protocols

Given the long study period and multicontinental setting, a detailed description of antibiotic regimens, adjunctive therapies (e.g., dexamethasone), and supportive care strategies is warranted. Differences in management could significantly influence mortality and may confound the associations observed.

7. CSF Leukocyte Sub-analysis

The analysis includes total CSF leukocyte counts but does not explore:

- Neutrophil vs lymphocyte predominance
- Neutrophil/lymphocyte ratio

There is clinical evidence that very high CSF neutrophil counts may correlate with worse outcomes. A sub-analysis considering cell differentials and pathogen type would be informative, especially given that some bacteria produce lymphocytic responses and prior antibiotic therapy can alter CSF profiles.

8. Effect of Pretreatment Antibiotics

Approximately 30–35% of patients received antibiotics prior to CSF analysis. Antibiotic therapy is known to alter CSF glucose, protein, and cellular profiles.

It would be important to clarify whether:

- The findings apply equally to pretreated and non-pretreated patients
- A subgroup analysis was performed stratified by prior antibiotic exposure

9. Need for Multivariable Regression Analysis

Multiple clinical and demographic factors influence mortality in bacterial meningitis. The current analysis is largely unadjusted.

A multivariable logistic regression model adjusting for:

- Age
- Region
- Pathogen
- GCS
- Seizures
- Pretreatment antibiotics
- Duration of illness

is necessary to determine whether CSF glucose and protein independently predict mortality.

10. Categorization of Continuous Variables

Several continuous variables were categorized into groups for analysis. While clinically intuitive, categorization reduces statistical power and may distort risk gradients.

Consider modeling CSF glucose and protein as continuous variables in regression analysis, potentially using spline modeling to assess non-linear effects.

11. Predictive Performance Metrics (ROC Curve)

The manuscript discusses “risk stratification,” yet no predictive performance metrics are presented.

Including:

- ROC curves
- Area under the curve (AUC)
- Sensitivity and specificity at key thresholds

would strengthen claims of prognostic utility.

12. Low CSF Leukocyte Count and Mortality

The statement that “children admitted with counts <500/mm³ had approximately a 50% greater risk of death” appears inconsistent with common clinical observations.

Further exploration is warranted:

- Were these patients immunosuppressed?
- Did they present late?
- Were specific pathogens overrepresented in this subgroup?

This finding requires deeper analysis and discussion.

13. Wide Confidence Interval in Combined Analysis (Figure 4)

The combined glucose/protein subgroup shows a very large odds ratio (OR 31.7) with a wide confidence interval (4.4–237), indicating limited precision and likely small sample size.

Absolute numbers should be clearly presented, and interpretation should be cautious.

14. Restriction to Bacteriologically Confirmed Cases

The study includes only confirmed cases. While this enhances diagnostic certainty, it limits applicability to real-world settings where many patients are culture-negative.

This limitation should be explicitly reflected in the conclusions, clarifying that findings apply only to confirmed bacterial meningitis.

15. Statement Regarding CSF-to-Blood Glucose Ratio

The statement suggesting practical limitations in measuring simultaneous blood glucose may be misleading. In routine practice, plasma glucose measurement at the time of lumbar puncture remains standard of care.

The findings of this study do not justify minimizing the importance of the CSF-to-blood glucose ratio in clinical practice, and this statement should be revised or removed.

7. PLOS authors have the option to publish the peer review history of their article (<https://journals.plos.org/plosone/s/editorial-and-peer-review-process#loc-peer-review-history>). If published, this will include your full peer review and any attached files. Do you want your identity to be public for this peer review? (Answer options: Yes, No): Yes

7a. If you answered "Yes" above, that you would like your identity to be public, please provide your full name, as it should appear on the published peer review. Please do not sign the review on behalf of another person.

Pramith Ruwanpathirana

Confidential to Editor:
1. Do you have any potentially competing interests? If none, type "None." Our policy on competing interests can be found at <http://journals.plos.org/plosone/s/competing-interests>.

None

Did you receive any assistance in preparing this review (e.g. from a post-doc or graduate student)? If yes, please provide their name and title. If you have chosen to reveal your identity to the authors in question 7 above, please include the name of the person who assisted you in the response to question 7 as well. (optional)

No

Do you want to receive recognition for this review on a Web of Science researcher profile? (<https://clarivate.com/products/scientific-and-academic-research/research-discovery-and-workflow-solutions/researcher-profiles/>)? If you opt in, your Web of Science profile will automatically be updated to show a verified record of this review in full compliance with the journal's review policy. If you don't have a Web of Science profile, you will need to first create a free account. (Answer options: Yes, No)

Yes
