# Supplementary material for: Early mortality risk stratification in childhood bacterial meningitis using cerebrospinal fluid glucose and protein levels and their combinations: A multicontinental cohort study
Source: PLoS One. 2026 Jun 9;21(6):e0351306. doi: 10.1371/journal.pone.0351306 (PMC13249170; doi:10.1371/journal.pone.0351306)
Supplement: Attachment — Submitted filename: Response to Reviewers.docx [file pone.0351306.s003.docx]

Dear Nilanka Perera,

Academic Editor, Plos One

Thank you for the opportunity to respond to the reviewers’ comments. Please find below our response letter, in which we have addressed the reviewers’ comments and implemented all requested revisions to the manuscript. We have also conducted the additional analyses suggested, where feasible.

In the final section of the letter, we provide our responses to the Editors’ comments as well.

Sincerely,

Markku Kallio, MD, PhD

**Reviewer #1:** This is a comprehensive and much-needed study with clear application to day-to-day practice.

Some minor revisions are suggested as below:

**Reviewer’s Comment:**
1. An indicator of the timing of the initial lumbar puncture and CSF analysis.

**Our response:** This was a large multicenter study conducted across six South American countries, Angola, and Finland, and precise timing data for the initial lumbar puncture and CSF analysis were unfortunately not recorded. However, across all participating hospitals, efforts were made to evaluate and manage patients with suspected meningitis as promptly as possible.

**Reviewer’s Comment:**
2. Clinically, the percentage drop in CSF glucose relative to the concurrent plasma glucose would be more helpful to the practicing clinician. Suggest a graph comparing the percentage of sugar drop with outcomes.

**Our response:** In many acutely ill children, glucose-containing intravenous fluids are initiated as soon as the child arrives at the emergency department. This can affect blood glucose measurements and may partly explain why the CSF-to-blood glucose ratio performed suboptimally in our dataset. In our view, a similar limitation would persist even when expressing the measure as the percentage decrease in CSF glucose relative to the concurrent plasma glucose. We revised our text of CSF glucose in Discussion.

**Reviewer #2**: I congratulate the authors for the excellent study which is clinically oriented and very useful for day to day practice. It is a large study with more than 1500 patients and is multi-centre. Here are some minor concerns which I would like to raise.

**Reviewer’s Comment:**

Description of Study Population (Latin America). It would improve clarity to explicitly list the individual Latin American countries included in the study and the number of participants recruited from each country. This will help readers better understand the geographic distribution and heterogeneity of the cohort.

**Our response:** The numbers of patients from each country have now been added to the manuscript in Methods

**Reviewer’s Comment**:

Very Long Study Period (1984–2017). The study spans 33 years, during which substantial changes have occurred in: Epidemiology of pathogens due to vaccination (Hib, pneumococcal vaccines), Antibiotic regimens and resistance patterns. Supportive and intensive care management, Diagnostic modalities (PCR availability, improved microbiology techniques).
 Pooling all data across this period may introduce temporal bias unless the authors demonstrate that pathogen distribution, management strategies, and outcomes were comparable across eras. A subgroup analysis by time period (e.g., pre- and post-vaccine eras) is strongly recommended.

**Our response:** The following answer is added to Results section: Because the study period was long, during which vaccines were introduced and treatment practices changed, we compared outcomes across each decade. The prognostic value of low CSF glucose and high CSF protein for mortality remained consistent across all time periods. Similarly, the findings were unchanged when analyses were stratified by the major bacterial pathogens.

**Reviewer’s Comment**:

Inclusion of Encephalitis Cases. Were patients with encephalitis included in this cohort? If so, please clarify the diagnostic criteria and describe their number and characteristics. If not, this should be clearly stated.

**Our response:** Only bacterial meningitis were included. This is added in material and methods.

**Reviewer’s Comment**:

Inclusion of Tuberculous Meningitis. Were patients with tuberculous meningitis included? If yes, please provide details. If excluded, this should be explicitly mentioned in the methods.

**Our response:** They were excluded. This is added in material and methods.

**Reviewer’s Comment**:

Breakdown of the “Other Bacteria” Category. The “other” pathogen group is heterogeneous. Certain organisms (e.g., Listeria monocytogenes, Mycoplasma pneumoniae) may cause lymphocytic predominance with less pronounced CSF glucose reduction or protein elevation but can still result in severe outcomes.
 A detailed breakdown of pathogens within the “other” category is essential. If numbers are small and heterogeneity substantial, consider A pathogen-specific sub-analysis. Or restricting conclusions to the major pathogens (H. influenzae, S. pneumoniae, N. meningitidis)

**Our response:** The ‘other bacteria’ group comprised 139 patients in total. The Cochran–Armitage test for trend remained statistically significant (p < 0.01). However, this group included 20 different bacterial species, resulting in very small numbers within each subgroup. Therefore, pathogen-specific trend analyses were not feasible due to insufficient statistical power

**Reviewer’s Comment**:

Description of Treatment Protocols. Given the long study period and multicontinental setting, a detailed description of antibiotic regimens, adjunctive therapies (e.g., dexamethasone), and supportive care strategies is warranted. Differences in management could significantly influence mortality and may confound the associations observed.

**Our response:** Given the long study period and multicontinental setting, treatment protocols varied across centers and over time, including differences in antibiotic regimens, use of adjunctive therapies (such as dexamethasone), and supportive care strategies. However, the prognostic value of low CSF glucose and high CSF protein for mortality remained consistent across these treatment variations. This is demonstrated in our aforementioned comparison between patients treated in different decades, and it is now reported in the Results section.

**Reviewer’s Comment**:
 CSF Leukocyte Sub-analysis. The analysis includes total CSF leukocyte counts but does not explore: Neutrophil vs lymphocyte predominance, Neutrophil/lymphocyte ratio. There is clinical evidence that very high CSF neutrophil counts may correlate with worse outcomes. A sub-analysis considering cell differentials and pathogen type would be informative, especially given that some bacteria produce lymphocytic responses and prior antibiotic therapy can alter CSF profiles.

**Our response:** Due to the multicenter nature of the study, conducted primarily in Africa and Latin America, CSF neutrophil/lymphocyte analyses were not consistently available.

**Reviewer’s Comment**:
 Effect of Pretreatment Antibiotics. Approximately 30–35% of patients received antibiotics prior to CSF analysis. Antibiotic therapy is known to alter CSF glucose, protein, and cellular profiles. It would be important to clarify whether the findings apply equally to pretreated and non-pretreated patients.

**Our response:** There were no differences between patients with and without antibiotic pretreatment; low CSF glucose and high CSF protein were equally significant predictors in both groups. This has been added to the Results section

**Reviewer’s Comment**:
 Need for Multivariable Regression Analysis. Multiple clinical and demographic factors influence mortality in bacterial meningitis. The current analysis is largely unadjusted. A multivariable logistic regression model adjusting for: Age, Region, Pathogen, GCS, Seizures, Pretreatment antibiotics, Duration of illness is necessary to determine whether CSF glucose and protein independently predict mortality.

**Our response:** We have added nominal logistic Regression analysis in the Result section

**Reviewer’s Comment**:
Categorization of Continuous Variables. Several continuous variables were categorized into groups for analysis. While clinically intuitive, categorization reduces statistical power and may distort risk gradients. Consider modeling CSF glucose and protein as continuous variables in regression analysis, potentially using spline modeling to assess non-linear effects.

**Our response:** We agree that continuous-variable modeling is statistically important and have therefore included multivariable analyses using CSF glucose and CSF protein as continuous predictors. However, our clinical objective was also to identify pragmatic cutoff values for routine bedside use. In emergency care, clinicians frequently interpret CSF glucose and CSF protein categorically as “markedly abnormal” or not, yet evidence-based thresholds for particularly dangerous values are lacking. We therefore present threshold-based analyses as a clinically complementary approach intended to provide practical support for early risk assessment.

**Reviewer’s Comment**:
Predictive Performance Metrics (ROC Curve). The manuscript discusses “risk stratification,” yet no predictive performance metrics are presented.
Including: ROC curves, Area under the curve (AUC), Sensitivity and specificity at key thresholds, would strengthen claims of prognostic utility.

**Our response:** The discriminatory performance of the final model was evaluated using receiver operating characteristic (ROC) curve analysis, and the area under the curve (AUC) is reported in the Results section. The model demonstrated acceptable accuracy in identifying patients at increased risk of mortality, supporting its potential utility for risk stratification. Notably, the concurrent presence of markedly decreased cerebrospinal fluid (CSF) glucose and elevated CSF protein levels was strongly associated with increased mortality risk, likely reflecting a pronounced inflammatory response.

**Reviewer’s Comment**:
Low CSF Leukocyte Count and Mortality. The statement that “children admitted with counts <500/mm³ had approximately a 50% greater risk of death” appears inconsistent with common clinical observations. Further exploration is warranted:
Were these patients immunosuppressed? Did they present late? Were specific pathogens overrepresented in this subgroup? This finding requires deeper analysis and discussion.

**Our response:** In severe infections such as meningitis with septic shock, CSF leukocyte counts may be unexpectedly low, reflecting overwhelming infection and impaired host response. Low CSF leukocyte counts have been shown to be independently associated with adverse outcomes in bacterial meningitis (references number 6 and a new reference number 27). This text has been added to Discussion.

**Reviewer’s Comment**:
Wide Confidence Interval in Combined Analysis (Figure 4). The combined glucose/protein subgroup shows a very large odds ratio (OR 31.7) with a wide confidence interval (4.4–237), indicating limited precision and likely small sample size. Absolute numbers should be clearly presented, and interpretation should be cautious.

**Our response:** The wide confidence interval reflects the small number of events in the reference group. Only one of 89 children with normal CSF glucose and protein levels died, whereas 44 of 122 children with markedly low CSF glucose and markedly elevated CSF protein died. The absolute values are shown in the figure.

**Reviewer’s Comment**:
Restriction to Bacteriologically Confirmed Cases. The study includes only confirmed cases. While this enhances diagnostic certainty, it limits applicability to real-world settings where many patients are culture-negative. This limitation should be explicitly reflected in the conclusions, clarifying that findings apply only to confirmed bacterial meningitis.

**Our response:** We edited the following sentence in Discussion in the Study Limitations section: Focusing on bacteriologically confirmed cases enhances reliability but may constrain generalizability to children with suspected meningitis and abnormal CSF findings without confirmed bacterial etiology.

**Reviewer’s Comment**:

Statement Regarding CSF-to-Blood Glucose Ratio. The statement suggesting practical limitations in measuring simultaneous blood glucose may be misleading. In routine practice, plasma glucose measurement at the time of lumbar puncture remains standard of care. The findings of this study do not justify minimizing the importance of the CSF-to-blood glucose ratio in clinical practice, and this statement should be revised or removed.

**Our response:** The reviewer is correct in noting that plasma glucose measurement at the time of lumbar puncture remains the standard of care. Our intention was to highlight the practical observation that many critically ill pediatric patients with meningitis had already been started on glucose-containing intravenous fluids in the emergency department prior to lumbar puncture. Consequently, in some cases, blood glucose measurements may have provided somewhat unreliable values. This may, in certain situations, further lead to distortion of the CSF-to-blood glucose ratio, potentially yielding somewhat inaccurate values. We revised our text in Discussion.

**Response to Academic Editor:**

**Academic Editor’s Comment**: If there are ethical or legal restrictions on sharing a de-identified data set, please explain them in detail (e.g., data contain potentially identifying or sensitive patient information:

**Our response:** The data underlying this study cannot be made publicly available due to ethical and legal restrictions. This study is a prospective, multicenter investigation conducted across eight countries on three continents, involving pediatric patients with meningitis. Ethical approval was obtained from the relevant Institutional Review Boards and Research Ethics Committees in each participating country and hospital prior to study initiation. The approved study protocols and informed consent procedures did not include provisions for unrestricted public data sharing in open-access repositories.

The dataset contains detailed clinical information on a vulnerable population (children with meningitis), collected across multiple international sites. Due to the combination of clinical variables, geographic information, and the relative rarity of certain presentations, there is a residual risk of re-identification. Furthermore, for participating sites within the European Union, data processing and sharing are subject to the General Data Protection Regulation (GDPR), which imposes strict requirements on the handling of personal health.

For these reasons, the ethics committees that approved the study have not granted permission for public deposition of the dataset. Data can be made available to qualified researchers upon reasonable request for scientifically valid purposes, subject to review and, where required, the relevant local ethics committees. Access will require a formal data use agreement to ensure compliance with applicable ethical, legal, and data protection requirements. Additional approvals from the originating site-specific ethics committees may be required prior to data sharing.

**Academic Editor’s Comment**: In the online submission form you indicate that your data is not available for proprietary reasons and have provided a contact point for accessing this data. Please note that your current contact point is a co-author on this manuscript. According to our Data Policy, the contact point must not be an author on the manuscript and must be an institutional contact, ideally not an individual. Please revise your data statement to a non-author institutional point of contact, such as a data access or ethics committee:

**Our response:** Here is the contact mail address to the Helsinki University Hospital (HUS) ethics committee

[eettinen.toimikunta@hus.fi](mailto:eettinen.toimikunta@hus.fi)
